# Storms & Neuralgia

**Published:** 1879-07

**Authors:** 


*Storms and Neuralgia.*—The *Sun* informs us that Dr. S. Weir Mitchell has discovered a new reason for the existence of the signal service and for the necessity of the storm signals issued by it. He read a paper before the National Academy of Sciences at its April meeting on "The Relation of Neuralgic Pain to Storms and the Earth's Magnetism." In the paper he recorded observations made by him in conjunction with Capt. Catlin, U. S. A., who, having lost a leg during the civil war, has since suffered from traumatic neuralgia in the stump. These observations include hourly records during five years, and the neuralgic attack was found to accord very nearly with the approach of a storm, and the pain to be greatest in the periods of greatest barometric depression. If these observations should be supported by others, so that the relation of neuralgic pain to storms can be established, we will find sufferers from the disease flying the world over from storm centres as faithfully as ships now do so at sea. When the danger signal is up the neuralgic patient who can't get away will retire to his home and send for his flannel wraps, his liniments and his doctor. He will look for the weather report in the paper in the morning before making up his mind to rise from bed, and will study it in the evening before going to his club, or to theatres and parties. The ladies, most of whom are neuralgic, will be afforded a new line of excuses for not seeing disagreeable and unwelcome visitors. "Pardon me," they can send down word, "the weather report announces that I am about to have an attack of neuralgia," or "Mrs. Jones desires to be excused under warnings from the signal service." These storm warnings will also be profited of by prudent husbands to dine or take a lunch down town. In fact the new system will work many reforms in our domestic relations.

